# Cognitive Functions and Cognitive Reserve in Relation to Blood Pressure Components in a Population-Based Cohort Aged 53 to 94 Years

**DOI:** 10.1155/2012/274851

**Published:** 2012-04-04

**Authors:** Nunzia Giordano, Valérie Tikhonoff, Paolo Palatini, Anna Bascelli, Giovanni Boschetti, Fabia De Lazzari, Carla Grasselli, Bortolo Martini, Sandro Caffi, Antonio Piccoli, Alberto Mazza, Patrizia Bisiacchi, Edoardo Casiglia

**Affiliations:** ^1^Department of Medicine, University of Padova, Via Giustiniani, 2-VIII Floor, 35128 Padova, Italy; ^2^Department of Geriatrics, Hospital of Schio, 36015 Schio, Italy; ^3^Department of Cardiology, Hospital of Thiene, 36016 Thiene, Italy; ^4^General Direction, Hospital of Verona, 37126 Verona, Italy; ^5^Nephrology Clinic, University of Padova, 35128 Padova, Italy; ^6^Department of Medicine, Hospital of Rovigo, 45100 Rovigo, Italy; ^7^Department of General Psychology, University of Padova, 35128 Padova, Italy

## Abstract

In 288 men and women from general population in a cross-sectional survey, all neuropsychological tests were negatively associated with age; memory and executive function were also positively related with education. The hypertensives (HT) were less efficient than the normotensives (NT) in the test of memory with interference at 10 sec (MI-10) (−33%, *P* = 0.03), clock drawing test (CLOX) (−28%, *P* < 0.01), and mini-mental state examination (MMSE) (−6%, *P* = 0.02). Lower MMSE, MI-10, and CLOX were predicted by higher systolic (odds ratio, OR, 0.97, *P* = 0.02; OR 0.98, *P* < 0.005; OR 0.95, *P* < 0.001) and higher pulse blood pressure (BP) (OR 0.97, *P* = 0.02; OR 0.97, *P* < 0.01; and 0.95, *P* < 0.0001). The cognitive reserve index (CRI) was 6% lower in the HT (*P* = 0.03) and was predicted by higher pulse BP (OR 0.82, *P* < 0.001). The BP vectors of lower MMSE, MI-10, and CLOX were directed towards higher values of systolic and diastolic BP, that of low CRI towards higher systolic and lower diastolic. The label of hypertension and higher values of systolic or pulse BP are associated to worse memory and executive functions. Higher diastolic BP, although insufficient to impair cognition, strengthens this association. CRI is predicted by higher systolic BP associated to lower diastolic BP.

## 1. Introduction

Dementia represents a considerable public health issue in developed countries due to progressive increase of affected individuals occurring with rapid ageing of population [1–3]. Actually, high blood pressure (BP) is one of the most important factors negatively affecting the modalities of cerebral aging [2–4]. Cerebral damage is mediated by changes in cerebral vasculature affecting both large and small vessels: the macrovascular atherosclerotic disease causes brain infarcts (either clinically evident as stroke, or silent), the microvascular disease results in chronic ischaemic changes affecting the white matter to a large extent [5, 6]. The outcome of single or multiple events is a stepwise progression to multi-infarct dementia, and that of chronic microvascular damage is a continuous progression from mild cognitive alterations to overt vascular dementia [5, 6].

The right approach to this problem is to place emphasis on preventive strategies to identify and counterbalance the risk factors at a population level.

Although hypoperfusion and neurodegeneration have emerged as possible underlying mechanisms, the pathophysiology of the relationship between high BP and low cognition remains unclear. Not only this, but also the BP levels that should be targeted to achieve optimal perfusion while preventing cognitive decline are still being debated [3–6]. Furthermore, it is uncertain if, to preserve cognition, it is better to keep low systolic BP, diastolic BP, or both.

The aim of the present study is to investigate the relationships of the different components of BP with cognitive function and cognitive reserve in a representative sample of general population and to identify the domains most affected.

## 2. Methods

### 2.1. Study Population

All men and women aged ≥50 years living in two Italian towns were invited by letter for a screening; 1,377 (76%) agreed with the study protocol, gave informed consent, and were recruited and regularly followed up; 288 randomly selected subjects (164 men and 124 women) underwent the neuropsychological tests described below and were considered for the analysis of data in the present work. Their general characteristics did not differ significantly from those of the remaining part of the sample (data not shown). The protocol of the LEOGRA study was extensively described elsewhere [7–9]. The study subjects, whose general characteristics are shown in Table 1, were seen by a staff of specialists at an *ad hoc* hospital unit, received a Rose's questionnaire about clinical history, smoking habits, and lifestyle, and underwent BP measurements in triplicate by trained medical doctors by means of an automatic 705 IT device (Omron Europe, Hoofddorp, Netherlands); the average of the last two measurements was taken into account for the analysis of data, and every effort was made to avoid terminal digit preference. Pulse pressure (PP) was defined as the difference between systolic and diastolic BP values.

### 2.2. Neuropsychological Assessment

Cognitive assessment was performed by means of mini-mental state examination (MMSE) [10] and a comprehensive neuropsychological battery of validated tests “paper and pencil” [11] relevant for exploring the areas of cognitive functions putatively related to cognitive decline.

Short-term memory was studied by means of digit span [12], long-term memory by means of immediate and delayed prose memory [11], and working memory with interference at 10 seconds [11]. The executive functions were explored using memory with interference at 30 seconds [11], the trail making test B [13], the phonemic verbal fluency test [14], and the Clock drawing test [15]. Attention was studied by means of the trail making test A [13] and of the overlapping figures [16].

The entire battery of tests was administered in a single session which took approximately two hours to complete. The digit span [12] consisted of memorization and repetition of a series of numbers. In immediate and delayed prose memory [11], a prose passage containing 30 words was presented to each participant on a one-to-one basis; immediate verbatim recalls were assessed, followed by a 10-minute delayed verbatim recall. In the tests of memory with interference at 10 and 30 seconds (MI 10 and MI 30, resp.), the participants were requested to recall a consonant trigram after an interval delays during which they had to count backward starting from a 3-digit random number presented by the examiner immediately after the trigram [11]. The overlapping figure was composed of 50 objects integrated into one perceptual unit [16]. Phonemic verbal fluency [14] required participants to generate appropriate names in a fixed period of time. In the trail making test A (TMT-A), subjects were required to connect with line progressive numbers, and in the TMT-B progressive numbers and letters [13]. In the clock drawing test (CLOX), the participant was instructed to draw a clock indicating 2 : 45 h, setting the hands and numbers on the face “so that a child could read them” [15]. The entire battery of tests was administered in a single session which took approximately 2 hours to complete.

The results of the neuropsychological battery were compared to the normative sample for Italian subjects aged ≥50 years [11]. This sample was also used to produce individual normal values for each test and to stratify subjects into those showing normal and impaired cognitive function.

Cognitive reserve index (CRI)—the ability to optimize and maximize performance through recruitment of brain networks and/or compensation by alternative cognitive strategies—was also measured through a validated questionnaire [17] to explore the difference between individuals in their capacity to cope with or compensate for pathology.

### 2.3. Definitions and Cut-off Values

The label of arterial hypertension [18] required systolic blood pressure ≥140 mmHg or diastolic blood pressure ≥90 mmHg or history of hypertension or appropriate antihypertensive treatment or hospital discharge with diagnosis-related group (DRG) 401–404 or 40200–40290 or 40300–40391. Body mass index (BMI) was calculated as the weight/squared height ratio, and obesity was defined as BMI ≥30 kg/m^2^. Truncal obesity was defined as suprailiac/triceps skinfold ≥2.24 in men or ≥1.32 in women [19].

Left ventricular hypertrophy required a left ventricular mass index ≥125 g/m^2^ in men or ≥110 g/m^2^ in women [20].

Subjects were labelled as diabetic when having fasting blood glucose repeatedly ≥126 mg/dL, blood glucose ≥140 mg/dL two hours after 75 g oral glucose, blood glucose ≥200 mg/dL at casual measurement, or current antidiabetic treatment confirmed by general practitioner [21]. As a measure of insulin resistance, the homeostasis model assessment index [22] was calculated from HOMA-R = (circulating insulin in *μ*U/mL) × (fasting blood glucose in mmol/L)/22.5.

History was positive for coronary artery disease when the Minnesota code was 1.2, 1.2, or 1.3 if absent 6.4.1, or 4.1 or 4.4 if absent 6.4.1, 7.1.1, and 7.2.1, or 5.1, 5.2, 5.3, or 5.4 if absent 6.4.1, 7.7.1, 7.2.1, and 7.4, or akinesia or dyskinesia were present at echocardiogram, or in the presence of positive myocardial scintigraphy or stress test, or of positive history of myocardial infarction or angina pectoris confirmed by hospital files, or in chronic appropriate antianginal treatment, or in the presence of hospital discharge with diagnosis-related group 410–414.

History was positive for cerebrovascular disease when in the presence of neurological signs, on positive history of stroke or transient ischaemic attack, or positive TC or MR or on hospital discharge with DRG 430–438.

Education was defined as years of schooling. MMSE was defined as low when scoring <24, MI-10, and CLOX when below the first tertile.

### 2.4. Statistical Analysis

Continuous variables were expressed as mean and standard deviation and compared with analysis of variance and the post hoc Bonferroni's correction. Categorical variables were expressed as percent rates and compared with the Pearson's *χ* ^2^ test. Multivariate regression analysis was used to identify the variables having a prognostic role on cognitive decline.

The label of “hypertension” was first used to stratify subjects, and the two categories (normotensive, hypertensive) were compared. The systolic and diastolic BP components were then used as independent variables in multivariate regression analyses having the test scores as dependent. Finally, as a unitary representation of the systolic and diastolic components [23], pulse pressure was used in the same multiple regressions.

Gender, age, BMI, historical cerebrovascular, and coronary events, education, arterial hypertension, diabetes, and antihypertensive treatment were used as covariables in multivariate analysis.

Finally, the joint distribution of mean systolic and diastolic BP was evaluated by BP vector analysis (mean vector with 95% confidence intervals) in subjects having normal or impaired test performance. This method has been described elsewhere [23–26] and is detailed in the Appendix. 

### 2.5. Ethical Considerations

The investigation met the principles outlined in the Declaration of Helsinki and institutional guidelines and was approved by the Local Ethics Committee. Before the study and after consulting his/her own general practitioner, each subject accepted and signed an informed consent.

## 3. Results

### 3.1. Descriptive Analysis of the Cohort

The cohort characteristics are shown in Table 1. Men represented 43% of the cohort, and age at examination was 73.5 ± 10.1 years (range 53 to 94). Years of education were on average 6.2 ± 2.6 (range 2 to 18).

No significant difference was detected between men and women. Consequently, the subsequent analysis was irrespective of gender.

### 3.2. Neuropsychological Tests

The scores of neuropsychological tests were not different from those expected for a normal group of reference persons of the same age [11]. In multiple regression analysis, all tests were related negatively with age. Prose memory, memory with interference, and executive function were also positively related with education (Table 2).

4After stratifying subjects according to the ESH/WHO label of hypertension [18] (Table 3), MMSE score was 6.2% lower in the hypertensives than in the normotensives. After adjustment for age and education, MI-10 and CLOX were performed less efficiently (−26% and −28%, resp.) in the former than in the latter. The other tests were carried out with comparable performance in the two groups.

In multiple regression analysis adjusted for age and education, both systolic BP and PP resulted to be independent predictors of MMSE, with MI-10 and CLOX, while the diastolic component taken alone did not (Table 4).

**Figure 1:**
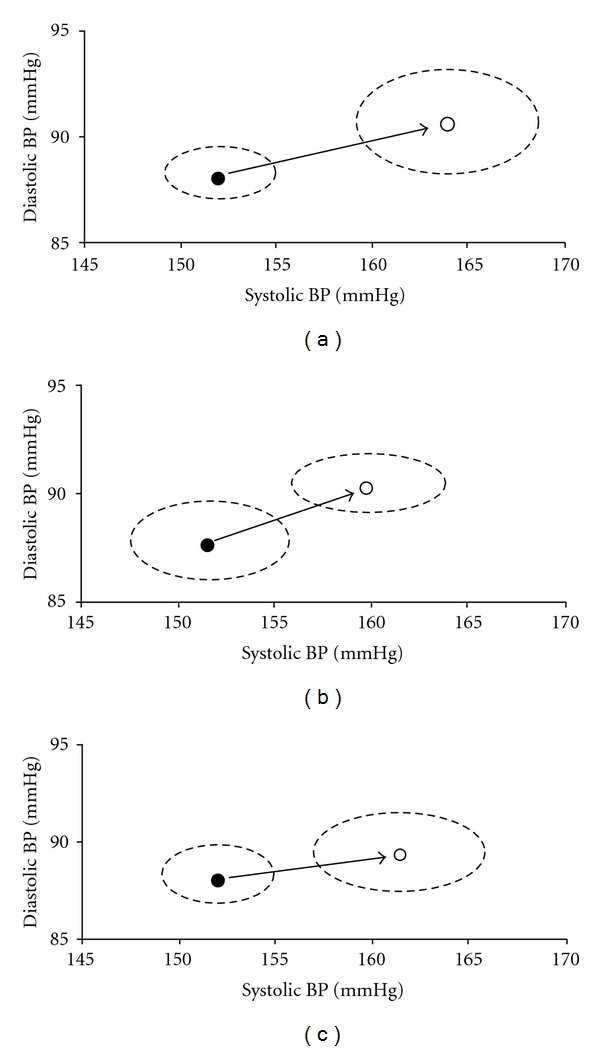
Vector analysis of the pair of variables “systolic blood pressure; diastolic blood pressure.” The points and the 95% confidence ellipses represent the mean values of systolic and diastolic pressure among subjects with normal (●) and low (∘) mini-mental state examination (a), memory with interference at 10 seconds, (b) and clock drawing test (c). The vector of impairment (arrow) is directed towards higher values of both systolic and diastolic blood pressure.

When systolic and diastolic BPs were analyzed together with bivariate vector analysis, the 95% confidence ellipses of the mean of the pair of variables “systolic BP; diastolic BP” obtained from subjects showing low MMSE, MI-10 and CLOX did not overlap (*P* nonsignificant) with those obtained from subjects showing normal performance; subjects with impairment were displaced towards higher systolic and higher diastolic BP values (arrow in Figure 1).

### 3.3. CRI

**Figure 2:**
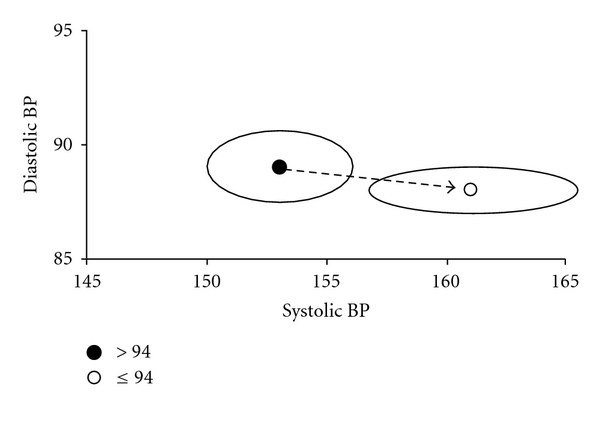
Vector analysis of the pair of variables “systolic blood pressure; diastolic blood pressure.” The points and the 95% confidence ellipses represent the mean values of systolic and diastolic pressure among subjects with cognitive reserve index ≥94 (●) and <94 (∘), the cut-off of 94 representing the median of this continuous variable for this cohort. The vector of impairment (arrow) is directed towards significantly higher values of systolic (+6 mmHg) and lower values of diastolic (−1 mmHg).

CRI was 6% lower in the hypertensives than in the normotensives (Table 3) and inversely predicted by PP in multiple regression model adjusted for age and education (Table 4). In bivariate vector analysis, the BP vector of impaired CRI was directed towards significantly higher values of systolic and lower values of diastolic BP (Figure 2), that is, higher values of PP.

## 4. Discussion

High BP is known to be a risk factor for cognitive decline, and many studies demonstrated a relationship between BP levels and cognitive impairment [27–30]. This association was confirmed in the present study, where high BP contributed to cognitive decline in a representative sample of general population of men and women. In our experience, this decline in subjects who were labeled as “hypertensive” according to current guidelines was not indiscriminate, but limited to working memory, executive functions, and global performance, while attention, language, visuospatial, and processing speed abilities were not affected. According to other authors [3], the systolic component of BP was associated to cognitive decline, while high diastolic BP *per se* was not and only acted as a factor able to increase the detrimental effect of high systolic values. In fact, diastolic BP was not an independent predictor in multivariate analysis, but (at least for the three tests shown in Figure 1) the BP vector of cognitive impairment was directed towards higher values of both systolic and diastolic.

The reasons of the association between arterial hypertension and impaired cognition are not completely understood, even though knowledge in this field is increasing. It is established that high BP causes directly or indirectly cerebral vascular damage (mainly *via* atherosclerosis in the larger vessels and oxidative stress in vascular wall [31]) and is also responsible for structural alterations in small-caliber vessels (particularly in the perforating arteries irrigating the cerebral white matter [32]). The macrovascular disease due to chronically high BP causes brain infarcts; the microvascular disease is associated to chronic ischaemic changes affecting the white matter and leading to multi-infarct dementia [33]. Not only this, but also it has been suggested that the vascular lesions accompanying high BP have a permissive effect on the clinical expression of Alzheimer's disease [34].

In our experience, the cognitive reserve was 6% lower in hypertensive than in normotensive subjects. The idea of a reserve against brain damage comes from the observation that the relationship between brain damage degree and clinical manifestation is not linear [35]. The cognitive reserve model suggests that the brain actively attempts coping with brain damage by using preexisting cognitive processing approaches or by enlisting compensatory approaches [36]. For this reason, a given brain damage can have different effects on different subjects, and individuals can sustain considerable brain damage before showing functional deficit [37]. Our impression is that high systolic and low diastolic BP have to coexist before the cognitive reserve becomes impaired. In fact, in our experience, CRI correlated inversely with PP, but not with systolic or diastolic BP taken separately. Furthermore, when systolic and diastolic were considered as a whole, the BP vector of lower values of CRI was directed towards higher systolic and lower diastolic, indicating that higher PP actually had a detrimental role on cognitive reserve greater than the systolic and diastolic components taken separately; +6 mmHg systolic and −1 mmHg diastolic were sufficient to stratify subjects into having lower or higher CRI.

In conclusion, our study contributes to the belief that a link exists between BP and cognition, higher values of systolic BP being associated to impaired cognitive function. Although high BP is not sufficient *per se* to reduce cognitive performance, higher values of diastolic BP are counterproductive in subjects with high systolic. This effect is not indiscriminate but limited to a set of functions and is probably mediated by impairment of vascular reserve and microvascular disease. Measures aimed at reducing or keeping low BP (particularly systolic BP) are mandatory in middle age to keep good cognitive abilities in late life. This underscores a need to consider pharmacological and nonpharmacological interventions to control arterial stiffening and maintain arterial compliance.

This study is novel in that it highlights the accessory role of diastolic in addition to systolic BP in modulating cognitive functions at a population level. Not only this, but also it clarifies that the cognitive decline accompanying high BP is not indiscriminate, but limited to memory and executive functions. Finally, it introduces in the estimation of cognitive decline an innovative concept, that of CRI, that seems to be particularly affected by pulse pressure.

## Figures and Tables

**Table 1:** General characteristics of the study cohort.

Body mass index (kg/m^2^)	27.0 ± 3.6
Obesity (%)	18
Left ventricular mass index (kg/m^2^)	123.9 ± 34.8
Left ventricular hypertrophy (%)	54.2
Systolic blood pressure (mmHg)	154.7 ± 21.3
Diastolic blood pressure (mmHg)	88.6 ± 9.2
Heart rate (bpm)	68.5 ± 8.7
Pulse pressure (mmHg)	66.1 ± 17.4
History of coronary events (%)	7.3
History of cerebrovascular events (%)	3.2
Haematocrit (%)	11.7 ± 9.6
Sedimentation rate (mm/h)	41.6 ± 3.4
Blood glucose (mg/dL)	103.9 ± 21.1
Diabetes (%)	18
Serum uric acid (mg/dL)	4.9 ± 1.3
Low-density-lipoprotein cholesterol (mg/dL)	159.4 ± 36.7
High-density-lipoprotein cholesterol (mg/dL)	46.3 ± 10.9
Serum triglycerides (mg/dL)	229.4 ± 43.5
Circulating cortisol (*μ*g/dL)	18.4 ± 4.8
Circulating T3 (ng/dL)	112.4 ± 21.8
Circulating T4 (*μ*g/dL)	8.5 ± 1.8
Plasma TSH (mlU/L)	1.8 ± 1.5
Circulating insulin (*μ*U/mL)	7.7 ± 5.5
HOMA-R	2.0 ± 1.7
Apolipoprotein B/A ratio	0.7 ± 0.2
Current smokers (%)	10.4
Cigarettes/day in smokers	10.5 ± 7.3 (median 9)

Mean ± standard deviation is provided for continuous variables, percent values for categorical variables. HOMA-R: homeostasis model assessment index; TSH: thyrotropin.

**Table 2:** Multiple regressions of the neuropsychological tests with age and education.

Tests	Age (years)		Education (years)	
	*b* (SE)	*P* value	*b* (SE)	*P* value
Mini-mental state examination	−0.14 (0.03)	<0.0001	−0.01 (0.09)	0.9 (NS)
Digit span	−0.03 (0.01)	<0.001	0.06 (0.03)	0.06 (NS)
Immediate prose memory	−0.19 (0.03)	<0.0001	0.38 (0.11)	<0.001
Delayed prose memory	−0.24 (0.04)	<0.0001	0.41 (0.13)	<0.001
Memory with interference at 10 sec	−0.08 (0.02)	<0.0001	0.20 (0.17)	<0.005
Memory with interference at 30 sec	−0.07 (0.02)	<0.0001	0.32 (0.06)	<0.0001
Phonemic verbal fluency	−0.12 (0.03)	<0.0001	0.28 (0.09)	<0.005
Trail making test A	0.93 (0.30)	<0.005	−2.78 (0.99)	<0.01
Trail making test B	2.45 (0.62)	<0.0001	−3.32 (2.04)	0.1 (NS)
Overlapping figure	−0.34 (0.06)	<0.0001	0.32 (0.20)	0.09 (NS)
Clock drawing test	−0.07 (0.03)	<0.01	0.27 (0.08)	<0.005

*b*: partial regression coefficient; SE: standard error of the coefficient; NS: nonsignificant.

**Table 3:** Cognitive tests scores by blood pressure value adjusted for age and education.

Tests	Whole cohort (*n* = 288)	Normotensive subjects (*n* = 54)	Hypertensives subjects (*n* = 234)	*P* value crude	*P* value adjusted for age and education
Mini-mental state examination	26.2 ± 3.7 (25.8–26.6)	27.6 ± 2.4 (27.0–28.3)	25.9 ± 3.9 (25.4–26.4)	<0.005	<0.05
Digit span	5.8 ± 1.3 (5.7–6.0)	6.2 ± 1.2 (5.8–6.5)	5.8 ± 1.3 (5.6–5.9)	0.04	0.3 (NS)
Immediate prose memory	8.4 ± 5.0 (7.8–9.0)	9.7 ± 5.6 (8.2–11.2)	8.1 ± 4.8 (7.4–8.7)	0.03	0.7 (NS)
Delayed prose memory	10.6 ± 5.9 (9.9–11.3)	12.6 ± 6.1 (11.0–14.3)	10.2 ± 5.8 (9.4–10.9)	<0.01	0.9 (NS)
Memory with interference at 10 sec	4.0 ± 3.1 (3.6–4.3)	5.4 ± 3.0 (4.6–6.3)	3.6 ± 3.0 (3.2–4.0)	<0.0001	<0.05
Memory with interference at 30 sec	1.2 ± 2.6 (0.9–1.5)	2.3 ± 3.3 (1.4–3.2)	1.0 ± 2.4 (0.7–1.3)	<0.005	0.2 (NS)
Trail making test A	77.0 ± 40.4 (71.8–82.1)	71.6 ± 41.9 (59.8–83.4)	78.4 ± 40.0 (72.7–84.2)	0.3 (NS)	0.6 (NS)
Trail making test B	158.1 ± 70.7 (144.8–171.5)	130.7 ± 59.3 (109.3–152.0)	169.4 ± 72.3 (153.1–185.7)	<0.01	0.2 (NS)
Phonemic verbal fluency	8.0 ± 3.9 (7.5–8.4)	9.0 ± 3.2 (8.2–9.9)	7.7 ± 4.0 (7.2–8.3)	0.03	0.9 (NS)
Overlapping figure	16.6 ± 8.5 (15.6–17.6)	20.1 ± 7.8 (18.0–22.3)	15.8 ± 8.5 (14.7–16.9)	<0.001	0.2 (NS)
Clock drawing test	6.4 ± 3.7 (6.0–6.8)	8.3 ± 2.6 (7.6–9.0)	6.0 ± 3.8 (5.5–6.5)	<0.0001	<0.01
Cognitive reserve index	94.5 ± 14.2 (92.5–96.4)	99.3 ± 9.9 (95.9–102.7)	93.5 ± 14.8 (91.2–95.7)	0.03	<0.05

95% confidence intervals in brackets; NS: nonsignificant difference.

**Table 4:** Regression of the test scores with the systolic and diastolic components of blood pressure.

	Systolic blood pressure		Diastolic blood pressure		Pulse pressure	
	*b*	*P* value	*b*	*P* value	*b*	*P* value
Mini-mental state examination	0.97 (0.95–0.99)	0.02	0.97 (0.91–1.03)	0.2 (NS)	0.97 (0.95–0.99)	0.02
Digit span	0.99 (0.98–1.00)	0.06 (NS)	0.99 (0.97–1.01)	0.5 (NS)	0.99 (0.98-0.99)	0.06
Immediate prose memory	0.99 (0.97–1.01)	0.6 (NS)	1.00 (0.94–1.06)	0.9 (NS)	0.99 (0.95–1.02)	0.5 (NS)
Delayed prose memory	0.99 (0.95–1.03)	0.6 (NS)	1.05 (0.99–1.11)	0.2 (NS)	0.97 (0.93–1.01)	0.1 (NS)
Memory with interference at 10 sec	0.98 (0.96–1.00)	<0.005	0.97 (0.93–1.01)	0.1 (NS)	0.97 (0.95–0.99)	<0.01
Memory with interference at 30 sec	0.99 (0.97–1.01)	0.2 (NS)	0.98 (0.95–1.02)	0.3 (NS)	0.99 (0.97–1.01)	0.3 (NS)
Trail making test A	0.95 (0.72–1.25)	0.7 (NS)	0.99 (0.98–1.00)	0.1 (NS)	1.16 (0.78–1.72)	0.4 (NS)
Trail making test B	1.31 (0.61–2.82)	0.5 (NS)	2.18 (0.50–9.56)	0.3 (NS)	1.09 (0.41–2.93)	0.8 (NS)
Phonemic verbal fluency	0.98 (0.96–1.00)	0.1 (NS)	1.00 (0.96–1.04)	0.9 (NS)	0.97 (0.94–1.00)	0.06 (NS)
Overlapping figures	0.99 (0.95–1.03)	0.5 (NS)	1.01 (0.91–1.11)	0.8 (NS)	0.97 (0.91–1.03)	0.4 (NS)
Clock drawing test	0.96 (0.94–0.98)	<0.001	0.99 (0.95–1.03)	0.8 (NS)	0.95 (0.93–0.97)	<0.0001
Cognitive reserve index	0.94 (0.87–1.02)	0.2 (NS)	1.09 (0.92–1.31)	0.4 (NS)	0.82 (0.73–0.92)	<0.001

95% confidence intervals in brackets; *b*: partial regression coefficients; NS: nonsignificant.

## Appendix

For readers less familiar with multivariate analysis, we report the basic definitions of multivariate confidence intervals as well as formulae for their calculations in the particular case of the assumption of the bivariate normal distribution for blood pressure vector (BP = SBP on *x* axis, DBP on *y* axis). Confidence interval is the inferential statistical interval for a given parameter (such as a mean value) investigated. It is the region in the parameter space to which is assigned the probability 100  (1 − *α*)% (*α* is some fixed probability, typically 0.05) that the parameter vector lies within. The confidence interval of the mean of the univariate normal distribution is formed by two values (limits), while the interval of the mean vector of the multinormal distribution is an ellipsoid centered at the mean vector, which reduces to a hypersphere when the correlation coefficients between pairs of variables are zero. When the confidence ellipsoids of two mean vectors overlap, the null hypothesis of equality of the two mean vectors cannot be rejected with the significance level *α* (i.e., nonsignificant Hotelling's *T* ^2^ test). The confidence interval becomes smaller with increasing sample size, and in a very large population, the confidence interval converges to the parameter vector (i.e., the mean vector point). The 95% confidence ellipses of several BP mean (bivariate) vectors are depicted in Figures 1 and 2.

Both approximate and exact methods are available for calculations of confidence ellipses of a bivariate normal distribution. Our modified version of the exact methods utilizes common statistics of the simple linear correlation analysis. Given *n* pairs of observations *x* and *y*, with standard deviations *s*_*x*_ and *s*_*y*_ and correlation coefficient *r*, and for a given probability level *α* (e.g., *α* = 0.05), take the Snedecor's *F*_*α*_ value with 2 and *n* − 2 degrees of freedom. The semiaxes *L*_1_ and *L*_2_ and the slopes *b*_1_ and *b*_2_ = − 1/*b*_1_ of the axes of the 100  (1 − *α*)% confidence ellipses can be calculated using (A.1) and (A.2), respectively,

(A.1) $$\begin{array}{cc} L1\;L2 & =\sqrt{K}\cdot \sqrt{\left(n-1\right)\left({s_{x}}^{2}+{s_{y}}^{2}\right)}\\ & \;+\sqrt{{\left[\left(n-1\right)\left({s_{x}}^{2}+{s_{y}}^{2}\right)\right]}^{2}}\\ & \;-4{\left(n-1\right)}^{2}\left(1-r^{2}\right)\;{s_{x}}^{2}{s_{y}}^{2}, \end{array}$$

where *K* = *F*/*n* · (*n* − 2) for confidence ellipses

(A.2) $$\begin{array}{cc} b,-\frac{1}{b} & =\frac{\left({s_{y}}^{2}-{s_{x}}^{2}\right)}{2rs_{x}s_{y}}\\ & \;\pm \sqrt{1}+{\left[\frac{\left({s_{y}}^{2}-{s_{x}}^{2}\right)}{2rs_{x}s_{y}}\right]}^{2}. \end{array}$$
